# Assessment of the inhibitory effects of disinfectants on the embryonation of *Ascaridia columbae* eggs

**DOI:** 10.1371/journal.pone.0217551

**Published:** 2019-05-29

**Authors:** Mohamed Bessat, Amira Dewair

**Affiliations:** Department of Parasitology, Faculty of Veterinary Medicine, Alexandria University, Alexandria, Egypt; UMASS Medical School, UNITED STATES

## Abstract

This study was set out with the aim of assessing the effects of the most commonly available commercial disinfectants on embryogenesis of *A*. *columbae* eggs. In addition to the distilled water treatment as a control group, four disinfectants were tested that included formalin, povidone iodide, TH4, and Virkon-S, in three independent experiments. In the first experiment, an overnight incubation with the working concentration of disinfectants resulted in significant inhibition of 80%, 85%, and 98% of embryonic development at day 9 post-treatment with formalin, povidone iodide, and TH4, respectively. This inhibition was continued through days 12 and 15 with the three tested disinfectants. Virkon-S did not affect embryogenesis with larval development comparable to that of the control group. In the second more dissected experiment, contact times of 10, 20, 30, and 60 minutes were set out for each disinfectant with embryogenesis inhibition results echoed that of the first experiment, with all disinfectants but Virkon-S inhibited larval development in significant proportions of eggs. Again, Virkon-S was very neutral in its effect on embryogenesis. When pigeon fecal matters were mixed with eggs and were subjected to disinfectants, discrepancies to results of the first two experiments were observed with only formalin inhibited embryogenesis in considerable proportion of eggs. Thus, and with the exception of Virkon-S, disinfectants tested at levels similar to those applicable in poultry in-houses and farms exhibited potent ovicidal activities on free eggs. To our knowledge, this is the first study involving the application of the commonly used in-poultry houses disinfectants to inactivate or delay the embryogenesis of bird Ascarids. The future perspective will potentially involve the in-field applications of the efficient disinfectants to eliminate or reduce the dissemination of infections with bird Ascarids in the chicken, turkey, pigeons, and other poultry houses.

## Introduction

Birds, both domestic and wild, are infected by a number of intestinal helminthes which belongs to classes of nematodes, cestodes, and trematodes[1]. Among them are the Ascarids of the order Ascaridida such as *Ascarida galli* and *Heterakis gallinae*, which affects most species of wild and domestic birds. While those two Ascarid species are getting the most attention when come to study bird’s helminthiasis, other species are as prevalent but receiving less attention. Examples are *Ascaridia dissimilis* in turkeys and *Ascaridia columbae* of pigeons. *Ascaridia columbae* (Gmelin, 1790) is the only enteric ascarid that infects the pigeon (Columba livia domestica) [2], with the life cycle and the morphology is well described after an experimental life cycle in pigeons [3,4].

Pigeons (Order Columbiformes) are ubiquitous birds and can be found in urban and rural areas in most towns and cities around the world [5]. Usually, pigeons are related to human and animal, they are kept as a protein source for human consumption, avocation and also for the experimental purposes [6].

*Ascaridia columbae* considered one of the most pathogenic parasites affecting pigeons causing ascaridiosis which can affect pigeon populations both in the low and high-density keepings [7]. Ascaridiosis usually occurs in cages and with breeding over land without the proper hygienic measures, conditions that greatly enhance infection dissemination among pigeon populations [8]. Infection with *A*. *columbae* is characterized by diarrhea, enteritis, and retardation in the growth rate [9]. Although symptomless in most cases, fatalities were recorded in some cases which were attributed to the rupture of the intestine occluded with worms and the associated peritonitis [10].

A common for *Ascaris* species that a single adult roundworm can lay thousands of eggs per day resulting in severe and rapid environmental contamination [11]. Moreover, *Ascaris* eggs are commonly showing high resistance to the adverse environmental conditions and may persist infective for months or even years in the human, animal, and birds dwellings [12]. Thus in order to limit the spread of these parasites, it is most important to prevent initial environmental contamination with eggs, which can only be achieved by implementing the appropriate cleaning, sanitation and disinfection measures. In this context, eggs of many *Ascaris* species proved highly resistant and can survive numerous chemical, biological and physical treatments such as strong bases and acids, protein-disrupting agents, oxidants, reductants, and surface-active agents [13]. Even the agents that are shown to be effective are either showing inconsistent results such as bleach, povidone-iodine, cresol, and alcohols [14–16] or are not readily applicable in the field applications such as UV radiations, ammonia, and hydrostatic pressure [17–19]. In two separate studies on *Ascaris suum* of pigs, 10% povidone iodine and some pesticides that are commonly used in agriculture were proved effective in inhibiting or delaying embryogenesis of *A*. *suum* eggs [20,21].

On the other hands, and when coming to bird Ascarids, no information is readily available on the efficacy of commonly applied disinfectants such as formaldehyde, bleach, and povidone-iodine against the common poultry *Ascaris* species. Thus and similar to the human and animal Ascarids, it is essential to limit the initial contamination of the environment by *A*. *columbae* eggs which can be achieved by the proper hygienic measures including disinfection. In this study, therefore, we evaluated the inhibition efficacy of some disinfectants commonly used in the poultry houses and farms on the embryonic development *A*. *columbae* eggs in the laboratory. Results were presented based on comparison of effects of initial concentrations, contact times and the ability to inhibit embryogenesis after a prolonged incubation time. Also, the inhibitory effect of disinfectants on eggs mixed with pigeon’s fecal matter was assessed.

## Material and methods

### Collection of *Ascaridia* worms and eggs

Adult *A*. *columbae* worms were collected from the naturally infected pigeons (*Columba livia domestica*) that were freshly dead, or were sacrificed for diagnosis purposes at the local Vet clinic. After removing worms from the small intestine, worms were washed three times in distilled water by centrifugation and re-suspension. Gravid adult females were selectively separated from male worms under the lower magnification of the dissecting microscope, which were then stored in distilled water at 5°C. To obtain eggs, gravid females were cut open into 3 parts and were then incubated at room temperature in distilled water for 30–60 minutes to release eggs. Released egg suspension was washed three times in distilled water by centrifugation (low speed) and re-suspension, before being applied in the designed experiments.

### Disinfectants preparation

Commonly applied disinfectants in the pigeon’s houses were applied as follow: Formalin 10% (by diluting the commercially available stock of 100% saturated formalin, 1/10 in distilled water), Povidone Iodine 10% (10 g Povidone Iodine in 100 ml distilled water), TH4 (THESEO, France) used as 2% as diluted from the stock solution in distilled water, while the disinfectant of Virkon S (Pharmacal, UK) was applied at 1% concentration (1 g in 100 ml distilled water). Distilled water was applied as a control group alongside the experimental groups of disinfectants.

### Treatment with disinfectants

Three experiments were performed to assess effects of disinfectants on *A*. *columbae* eggs, with each experiment was technically replicated in duplicates.

In the first experiment, the long-term effects of different disinfectants were assessed. About 2000 eggs were mixed with a 1-ml aliquot of each of distilled water (Control group G1) and with each of four disinfectants (G2-G5) in sterile polystyrene 15-ml falcon tubes. Control and treated groups were prepared in duplicates and were further incubated at room temperature through overnight (a contact time mainly applied in cleaning birds’ facilities). Disinfectants were removed by washing, centrifugation (low speed), and the re-suspension in distilled water repeated for 3 times. The treated and control groups were further incubated for additional 15 days, which is the average period required for embryonic developments in *A*. *columbae* species [3,4].

In the second experiment, the effects of contact times with different disinfectants were determined. In doing this, 4 sub-groups of egg mixture with each of 4 disinfectants were prepared as above and were subjected to different treatment (contact) times (10, 20, 30, 60 min) at room temperature. After each treatment time, disinfectants were removed by washing and centrifugation as above. Different sets of egg suspensions were mixed with 1-ml aliquots of distilled water in sterile polystyrene 15-ml falcon tubes. All groups were further incubated at room temperature for 15 days to allow fully embryonated larvae to develop inside eggs.

The third experiment was performed to assess the effects of disinfectants on pigeon’s fecal matter tainted with *A*. *columbae* eggs. ~ 250 grams of fecal matter collected from pigeon’s house was autoclaved (one cycle, 121°C, 20 min). This is then divided into 5 pools of 50 gram each in autoclaved, small glass beakers. About 2000 eggs were mixed with a 5-ml aliquot of each of distilled water (G1) and with each of four disinfectants (G2-G5). Egg mixtures were them mixed with fecal matter in glass beakers by using clean spatulas. All five groups prepared in duplicates were further incubated at room temperature for an additional 15 days. To avoid dryness effect and to keep enough humid environments for eggs development, a 5-ml aliquot of distilled water was added to each group at 72-hours intervals. Processing of fecal samples for the recovery of ova was done according to the methods of [22] and [23], with some modifications. 50 g of fecal-egg sample was taken in a 50 ml falcon tube, was soaked overnight in tap water, and contents were mixed thoroughly in the tube for ten minutes. The supernatant was discarded and saturated sodium chloride solution (NaCl) was added until tubes were topped to its meniscus surfaces. Later a coverslip was touched on the meniscus and placed on a microscopic slide.

### Assessment of the embryonic developments

In the first two experiments, embryonic developments inside eggs were assessed at three different time points (9, 12, and 15 days) post-treatment. In the third experiment, eggs were only assessed at the end of the 15-day time course. Eggs were assessed for development under ×100 magnification of binuclear light microscope (Optika, Italy). This is done by transferring the sample aliquot of each treatment (1-ml in experiments 1&2) to a 3-cm-diameter clear plastic petri-dish, which is divided into cm^2^ squares by a fine-point marker pen in a custom way. The total volume of each sample was counted; with the sample has to be divided into several aliquots to facilitate the counting process. In experiment 3, eggs were assessed by examining the coverslip placed on a microscopic slide.

A total of about 100 eggs were observed at each time point with only those eggs having a clear, dividing embryonic cell or fully developed larva was counted as viable eggs. Each count process was repeated in triplicates (3 times) for each assessed sample. The survival rate in each experimental group was calculated based on the following equation: Survival rate (%) = Number of viable eggs/total number of eggs*100, with the mean standard deviation (±SD) values were calculated out of the triplicate data. Significance of variations observed among different groups was evaluated by using the Analysis of Variance ANOVA (SPSS, USA), with significant differences were considered with a *p*-value of <0.05.

## Results

**Fig 1:**
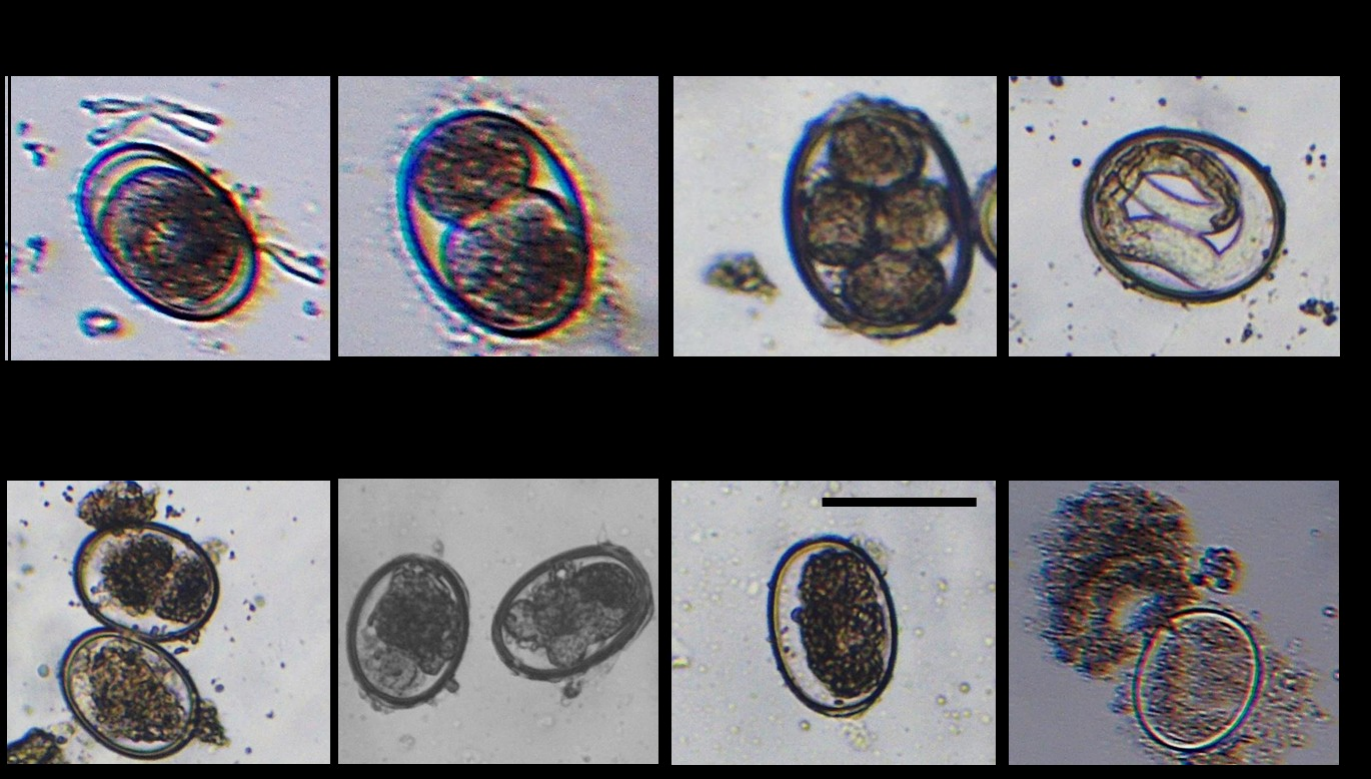
Morphologic-microscopic findings of development of A. columbae under the control conditions (the upper panel); and after disinfectants treatment (the lower panel). The upper panel shows various developmental stages of embryo from egg with one-cell stage (left), two-cell stage egg (second left), four-cell stage egg (third left), leading to the egg with fully developed larva (right). The lower panel demonstrates the various anomalies recorded after disinfectants as eggs with disintegrated early embryo (left), eggs with disintegrated late embryo (second left), egg with a dead larva (third left), and even a disintegrated (ruptured) egg (right). The scale bar represents 70 μM.

After removal from the naturally infected pigeons, adult worms were identified microscopically to the species level of *A*. *columbae* based on the characteristic morphological keys at the genera and species levels [2,4]. Although the main study concerning the developmental life cycle of *A*. *columbae* did not embark on details of embryonic development inside eggs but instead it only documented the development of larval stages (L1 to L4) at 12 to 15 days period [4]. Nevertheless, in our study, we did observe embryonic development at an earlier point (day 9) at which we identified the dividing embryo that featured the two and four-cell stages (Fig 1, upper panel), in addition to viable larvae. On the other hand, signs of non-viability included eggs with fragmented non-dividing embryonic cells, eggs with dead larvae, and eggs with ruptured shell (Fig 1, lower panel).

In the first experiment with an overnight incubation with disinfectants, clear discrepancies were observed between different groups (Table 1). In the control group and at the day 9 post-incubation, 75% of eggs were identified with marked embryonic development with most of the eggs featured two and four-cell stages, with many eggs identified microscopically with viable larvae (Fig 1 upper panel; Table 1). The percentage of eggs with viable embryo increased to 80% and 78% on days 12 and 15, respectively. When compared with the control group, all the disinfectants but Virkon-S showed marked inhibitory effects on embryonic development of *A*. *columbae* eggs which is very evident by the marked reduction in the number of viable eggs at the day 9 post-treatment (Table 1). The most significant inhibition occurs with TH4 with only 2% and 5% of eggs were identified with viable embryo at 9 and 12 days, respectively, while not even a single egg with viable embryo was evident at day 15 post-exposure. Similar to TH4 inhibition albeit to a lesser extent were formalin and povidone iodine which inhibited embryonic development in more than 75% and 85% of eggs, respectively (Table 1). Interestingly and to our surprise was the result of Virkon S treatment which produced efficient embryonation even better than that of the control group (80–95% compared with 75–80%, respectively). Among deficient embryogenesis observed after disinfectants exposure, the most observed anomalies were eggs with disintegrated early embryo, eggs with a disintegrated late embryo, and eggs with dead larva (Fig 1). A considerable number of eggs lost their intactness (ruptured), specifically in the two disinfectant groups of formalin and TH4 after the 15-day incubation period.

**Table 1:** Effects of different disinfectants on embryonic larval development of *A*. *columbae* eggs.

Group	Disinfectant	Conc.	0-day	9-day	12-day	15-day
**G1**	**Control**	Dist. H2O	100	75±0.3	80±1.0*	78±1.8
**G2**	**Formalin**	10%	100	20±0.6*	30±0.6*	24±0.6
**G3**	**Povidone Iodine**	10%	100	15±1.0*	20±1.0*	12±1.2
**G4**	**TH4**	2%	100	2±0.6*	5±1.0*	0±1.0*
**G5**	**Virkon S**	1%	100	80±0.4*	95±0.8	90±1.0*

Data are presented as percentages of viable eggs out of 100 counted eggs

± reports the standard deviation (STDEV) values out of the three count replicates of each treatment

* p-values of <0.05 out of the repeated measures ANOVA

Because an overnight incubation with disinfectants appeared too long to be practiced in the field conditions, a dissecting second experiment was set out to detect the variant inhibitory effects with different disinfectants. In doing that, ascending contact times of 10, 20, 30 and 60 min were set for each disinfectant. Results, as shown in Table 2, echoed those observed above for the fixed contact time, with various degrees of inhibitory effects that corresponded well to the contact times. As similar to the first experiment, each of formalin, povidone iodide, and TH4 inhibited larval development, with inhibitory effects from day 9 through 12 and 15 days post-treatment. Again, TH4 was the most effective with more than 75% of eggs with inhibited embryogenesis at the earliest point of day 9 and with the minimum contact time of 10 minutes. At similar time points and contact times, formalin and povidone iodide inhibited embryogenesis in 50% and 58% of eggs, respectively. A prolonged contact time of 60 minutes resulted in greater inhibition percentages of 75%, 85%, and 75%; with formalin, povidone iodide, and TH4, respectively (Table 2). With the three disinfectants at 60-minute contact times, 12 and 15-days-time points see aggravated inhibition in larval development with only 7%, 18%, and 8% of eggs were detected with viable larvae at the 12-day time point, while no discrete (intact, countable) eggs were detected when waiting up to day 15. Again, and regardless of the incubation time that was tried, Virkon-S was totally ineffective in inhibiting embryogenesis with more than 80% of eggs with viable larvae could still be detected at day 15 post-treatment.

**Table 2:** Time-points inhibitory effects of different disinfectants on embryonic larval development of *A*. *columbae* eggs.

Group	Disinfectant	Conc.	Contact time (min)	0-day	9-day	12-day	15-day	p-value
**G1**	**Control**	Dist. H2O	0	100	60±0.2	78±0.2	83±0.6	<0.05
**G2a**	**Formalin**	10%	10	100	50±0.4	25±1.2	20±0.8	-
**G2b**	**Formalin**	10%	20	100	35±1.0	20±1.2	20±1.2	<0.05
**G2c**	**Formalin**	10%	30	100	20±1.2	12±0.6	Undetectable	<0.05
**G2d**	**Formalin**	10%	60	100	25±0.1	7±0.1	Undetectable	<0.05
**G3a**	**Povidone Iodine**	10%	10	100	42±1.2	50±1.2	43±1.0	-
**G3b**	**Povidone Iodine**	10%	20	100	25±0.4	40±0.4	30±1.2	<0.05
**G3c**	**Povidone Iodine**	10%	30	100	30±0.7	20±1.0	8±0.8	<0.05
**G3d**	**Povidone Iodine**	10%	60	100	15±1.4	18±0.8	5±1.0	<0.05
**G4a**	**TH4**	2%	10	100	24±0.8	30±1.2	20±1.2	<0.05
**G4b**	**TH4**	2%	20	100	30±1.0	28±0.2	Undetectable	<0.05
**G4c**	**TH4**	2%	30	100	22±0.8	10±0.7	Undetectable	<0.05
**G4d**	**TH4**	2%	60	100	25±1.2	8±1.2	Undetectable	<0.05
**G5a**	**Virkon S**	1%	10	100	80±1.3	87±1.3	90±1.3	<0.05
**G5b**	**Virkon S**	1%	20	100	83±0.7	85±0.7	87±0.7	<0.05
**G5c**	**Virkon S**	1%	30	100	80±0.6	80±0.6	90±0.6	<0.05
**G5d**	**Virkon S**	1%	60	100	80±1.2	82±1.2	80±1.2	<0.05

Data are presented as percentages of viable eggs out of 100 counted eggs

± reports the standard deviation (STDEV) values out of the three count replicates of each treatment

The third experiment was set out to assess inhibitory effects with different disinfectants on pigeon’s fecal matters that were experimentally soiled with *A*. *columbae* eggs. Except formalin, all the three disinfectants have an unequivocally very weak inhibitory effect that was comparable to the control group (Table 3). Formalin was the only disinfectant with effective inhibitory effect on eggs in fecal matter with only 35% showing signs of embryogenesis at day 15 post-treatment.

**Table 3:** Assessment of inhibitory effects of different disinfectants on *A*. *columbae* eggs-tainted fecal matter.

Group	Disinfectant	Conc.	15-day
**G1**	**Control**	Dist. H2O	78±1.5
**G2**	**Formalin**	10%	35±2.0*
**G3**	**Povidone Iodine**	10%	72±1.5
**G4**	**TH4**	2%	75±1.5*
**G5**	**Virkon S**	1%	75±0.8*

Data are presented as percentages of viable eggs out of 100 counted eggs

± reports the standard deviation (STDEV) values out of the three count replicates of each treatment

* p-values of <0.05 out of the repeated measures ANOVA

## Discussion

Different disinfection protocols have been practiced by owners of animal and birds facilities at their farms and houses; which led us to the idea of trying the most traditional disinfectants applicable in the pigeon houses in order to control one of the most common enteric parasites of pigeons, *A*. *columbae*. Our data have indicated that most agents widely used in disinfecting pigeon houses are generally effective in inhibiting or at least minimizing the dissemination of Ascarid eggs between bird hosts. While the biological development and the life cycle features of *Ascaridia* species of pigeon and that of chicken and turkey are very similar, we perhaps can presume that the same disinfectants would be effective in the chicken and turkey breeding houses too. Nevertheless, a future similar research would be then required to confirm this anticipation. The dual use of these disinfectants as anti-microbial and now as anti-parasitic is of economic value by reducing the costs required as if the two disinfection protocols (microbial and parasitic) were to be practiced independently.

Comparison of effects of disinfectants on Ascarid eggs of birds (this study) and those observed with Ascaris species of human and animal hosts generally revealed that while disinfectants are mostly detrimental to embryogenesis in the former case, they are generally ineffective in the latter case[17,24,25]. Also while the povidone iodine is generally effective in our case by inhibiting embryogenesis at the shortest contact time of 10 minutes, there were conflicting reports of its effectiveness on the human and animal *Ascaris* eggs as while high efficacy has been reported in some studies [15,16], complete ineffectiveness has been reported in other studies [14,20]. The only treatment that has been reported to totally inactivate Ascaris eggs in minutes is by heating above 60°C[26], a condition that is technically inapplicable in the in-house and in-farms disinfection procedure. The starkest contrast comes with the use of quaternary ammonium compound which is completely ineffective in inhibiting the development of *A*. *suum* eggs [14], while here in this study is highly efficient in deactivating *A*. *columbae* eggs as represented by TH4 which contains quaternary ammonium chloride compound.

Discrepancies observed between eggs of human and animal Ascaris and these of birds Ascarids is arguably explained in the light of structural differences as while the former eggs are corticated (shell covered with thick conspicuous mamillation), those of bird Ascarids are thin-shelled eggs without mamillation [27].

When dissecting data about the efficacy of different disinfectants, the potent ovicidal activity of povidone iodide is in total agreement with that reported for *A*. *suum* eggs in which the 10-minute exposure to 10% concentration was able to kill more than 95% of eggs [20]. The second disinfectant, formalin with no surprise turned to be highly efficient in killing ≥80% of eggs at days 9 and 12 with no eggs detectable at day 15 post-treatment. The third and the most ovicidal efficient agent out of the four tested disinfectants turned to be TH4. TH4 has been reported as an effective agent against a variety of pathogens that included bacteria, viruses, and fungi (URL1). In specific viral disease outbreaks such as adenovirus, paramyxovirus, poliovirus and picornavirus, the disinfectant is applied at 1:100 to 1:50 dilution (1–2%), while its application at 1:200 (0.5%) is applied with no specific disease cases. Its application at 2% in this study proved to be highly effective in inhibiting development even with the least contact time of 10 minutes. This corresponded well with the great reputation of TH4 as the ideal disinfectants in the pigeon houses and farms, not only due to its high efficacy as a virucidal agent but now as a potent ovicidal disinfectant.

While it will be ideal to try disinfectants under field conditions in pigeon houses, this is technically very difficult as it is almost impossible to obtain fecal matter with uniform natural infection intensities. Thus, we have tried to simulate field conditions by mixing eggs with fecal matter in the third experiment. Nevertheless, only formalin proved efficient in affecting embryonic development. The most plausible explanation of the absence of inhibitory effects with other three disinfectants most likely originates from fecal matter, with organic components in feces which most likely bind to disinfectants and counteracts their effects, rendering it highly ineffectual in field situations.

When compared together, some similarities and discrepancies were detected between four applied disinfectants in regard to their effectiveness, time required to achieve inhibitory effects, and the observed destructive effect on eggs.

What is common between formalin and TH4 plus their embryogenesis inhibiting effect, is their ova destroying effect with no intact eggs were ever detectable at day 15 post-treatment. When looking at the chemical structure, both agents contain aldehydes which are formaldehyde in the formalin and glutaraldehyde in TH4 (URL2), which might explain the efficient ova destructive effect shared between both agents.

On contrary to the efficient embryogenesis inhibiting effect of the three agents of formalin, povidone iodide and TH4, Virkon-S-treated group not only behalf as the control group (distilled water) with no effect on eggs development but it also enhances embryogenesis with a higher percentage of eggs showing larval development than the control group at the end of the treatment experiment (day 15). This is cannot be simply explained by looking at the physiochemical properties of Virkon-S. Virkon is a multi-purpose disinfectant as it contains oxone (potassium peroxymonosulfate), sodium dodecylbenzenesulfonate, sulfamic acid, and inorganic buffers. It is typically used in hospitals, laboratories, and veterinary facilities, as the agent has a wide spectrum of activity against viruses, some fungi, and bacteria[28]. However, it is less effective against spores and fungi than some alternative disinfectants [29], which might help to explain the absence of any ovicidal effect for the Virkon-S in the current study.

## Conclusion

Summarizing the results of the present study has concluded that some of the commonly applied disinfectants in the pigeon houses were proved very efficient in inhibiting the embryogenesis of *A*. *columbae* eggs. TH4 proved itself as an indispensible disinfecting agent in poultry houses as a dual agent against microbial and parasitic infections. Similarly, the traditional disinfectants of formalin and povidone iodide remained valid also as dual anti-microbial and anti-parasitic agents. Nevertheless, mechanisms behind the ovicidal activity of these disinfectants require further research. More importantly, it will be still interesting to carry out future research looking into the potential of disinfectant-treated *A*. *columbae* eggs to infect and develop inside pigeon hosts. Due to its apparent inefficacy, a proper anthelminthic protocol is recommended when Virkon-S is in effect.
